# Vaginal Flora in Postmenopausal Women: The Effect of Estrogen Replacement

**DOI:** 10.1155/S1064744993000225

**Published:** 1993

**Authors:** Paul D. Ginkel, David E. Soper, Richard C. Bump, Harry P. Dalton

**Affiliations:** 1 Department of Obstetrics and Gynecology Medical College of Virginia Virginia Commonwealth University Richmond VA USA; 2 Department of Pathology Medical College of Virginia Virginia Commonwealth University Richmond VA USA; 3 Box 34 MCV Station Richmond VA 23298 USA

## Abstract

*Objective:*To determine the effect of estrogen replacement therapy (ERT) on the vaginal flora of postmenopausal women.

*Methods:* Vaginal cultures were obtained from 15 postmenopausal women whose hormonal statuses were documented by serum follicle-stimulating hormone (FSH) and serum estrogen levels. After 8 weeks of ERT, consisting of 0.1 mg of estradiol delivered daily by dermal patch, the vaginal cultures were repeated, as were measurements of the vaginal pH, serum FSH, and serum estrogen levels.

*Results:* Vaginal cultures revealed no significant change in the incidence of lactobacilli or of all aerobes. However, the incidence of anaerobic species fell after treatment from 47% to 13% (*P* = 0.05), and the incidence of anaerobic gram-negative rods declined after treatment from 40% prior to ERT to 7% (*P* = 0.035). Prior to ERT, the difference in mean vaginal pH between lactobacilli-positive and lactobacilli-negative subjects was not significant, but, following the administration of exogenous estrogen, the lactobacilli-positive subjects exhibited a significantly lower mean vaginal pH (4.4 ± 0.4) relative to the lactobacilli-negative population (5.2 ± 0.3) (*P* = 0.02).

*Conclusions:* We conclude that women on ERT are less likely to have vaginal colonization with anaerobic bacteria when compared with women not using replacement therapy. Estrogen replacement may potentiate the effect of lactobacilli on vaginal pH.


Infectious Diseases in Obstetrics and Gynecology 1:94–97 (1993)
© 1993 Wiley-Liss, Inc.

### Vaginal Flora in Postmenopausal Women: The Effect of Estrogen Replacement

**Paul D. Ginkel, David E. Soper, Richard C. Bump, and Harry P. Dalton**

*Departments of Obstetrics and Gynecology (P.D.G., D.E.S., R.C.B.) and Pathology (H.P.D.), Medical College of Virginia, Virginia Commonwealth University, Richmond, VA*

Abstract

***Objective:*** **To determine the effect of estrogen replacement therapy (ERT) on the vaginal flora of postmenopausal women.**

***Methods:*** **Vaginal cultures were obtained from 15 postmenopausal women whose hormonal statuses were documented by serum follicle-stimulating hormone (FSH) and serum estrogen levels. After 8 weeks of ERT, consisting of 0.1 mg of estradiol delivered daily by dermal patch, the vaginal cultures were repeated, as were measurements of the vaginal pH, serum FSH, and serum estrogen levels.**

***Results:*** **Vaginal cultures revealed no significant change in the incidence of lactobacilli or of all aerobes. However, the incidence of anaerobic species fell after treatment from 47% to 13% ($P = 0.05$), and the incidence of anaerobic gram-negative rods declined after treatment from 40% prior to ERT to 7% ($P = 0.035$). Prior to ERT, the difference in mean vaginal pH between lactobacilli-positive and lactobacilli-negative subjects was not significant, but, following the administration of exogenous estrogen, the lactobacilli-positive subjects exhibited a significantly lower mean vaginal pH (4.4 ± 0.4) relative to the lactobacilli-negative population (5.2 ± 0.3) ($P = 0.02$).**

***Conclusions:*** **We conclude that women on ERT are less likely to have vaginal colonization with anaerobic bacteria when compared with women not using replacement therapy. Estrogen replacement may potentiate the effect of lactobacilli on vaginal pH.** © 1993 Wiley-Liss, Inc.

Key words

Estrogen, vaginal flora, postmenopausal

In order to examine the effect of hormonal status on the vaginal flora, previous investigators have compared bacterial isolates of premenopausal and postmenopausal women. Tashijian et al. noted a higher incidence of aerobic gram-negative rods in postmenopausal women; however, the hormonal status of the subjects was never documented.[1] Although a subsequent study revealed no significant difference between the bacterial isolates obtained from premenopausal and postmenopausal populations, some of the subjects in the postmenopausal population were receiving estrogen replacement therapy (ERT) and, again, the hormonal status of the subjects was not documented by any objective parameter.[2] In contrast, a more recent study by Larsen et al. used vaginal cytology to document menopausal status and compared vaginal bacterial isolates obtained from postmenopausal women receiving ERT with the isolates obtained from a separate postmenopausal population not receiving exogenous estrogen.[3] This study found no significant difference in the incidence of aerobic organisms, although anaerobic species were more frequently isolated in the untreated population.

The purpose of the present investigation was to compare the vaginal microbial flora in postmeno-

**Address correspondence/reprint requests to Dr. David E. Soper, Box 34, MCV Station, Richmond, VA 23298.**

Received April 28, 1993

Accepted July 20, 1993

Clinical Study pausal women prior to (the control group) and 8 weeks after beginning ERT (the study group).

### MATERIALS AND METHODS

The subjects used in the study were 15 postmenopausal outpatients of Medical College of Virginia hospitals who were eligible for ERT and whose postmenopausal status was confirmed by serum estrogen and follicle-stimulating hormone (FSH) levels. At the time of the patient's initial visit, a complete gynecological history was obtained, detailing the menstrual, reproductive, contraceptive, and sexual history of the subject as well as accounting for recent antibiotic exposure and significant underlying disease. Thereafter, a complete gynecological physical examination was performed including a test of vaginal pH using ColorpHast pH test strips (EM Science, Cherry Hill, NJ). A vaginal washing was obtained using 3 ml of sterile, nonbacteriostatic saline that was instilled into the vagina, agitated with a sterile swab, and aspirated into a syringe. After excess air was expelled, the syringe was capped and transported immediately to the laboratory.

ERT consisted of estrogen delivery by means of a patch providing 0.1 mg of estradiol per day. Following 8 weeks of ERT, the assessment detailed above was repeated including a repeat sampling of serum estradiol and FSH levels.

Anaerobic cultures of the vaginal washings were incubated in an anaerobic chamber for 7 days using reduced brucella-base blood agar with 10 μg/ml of menadione and 0.5 μg/ml of hemin (BMB), BMB with 75 μg/ml of kanamycin and 7.5 μg/ml of vancomycin, and BMB containing 100 μg/ml of neomycin sulfate. Media for the isolation of aerobes and facultative anaerobes included 5% sheep's blood agar (BAP), MacConkey agar, and Pfizer Selective Enterococcus agar (PSE). These were incubated in either air (BAP) or in 5% carbon dioxide (MacConkey agar and PSE). All organisms were identified by the standard methods previously outlined.[4]

Differences in the number of positive cultures obtained before and after ERT were tested for significance using a Chi square or Fisher's exact test. Quantitative data including vaginal pH, serum FSH, and serum estradiol were tested using a two-tailed t test. All *P* values were based on two-tailed tests with a $P \leq 0.05$ considered significant.

**TABLE 1. Population characteristics before and after estrogen replacement therapy (ERT)**

| Variable | Pre-ERT[a] | Post-ERT[a] | *P* value |
|---|---|---|---|
| Serum FSH (mU/ml) | 144.3 ± 58.6 | 67.9 ± 28.0 | 0.0002 |
| Serum estradiol (mU/ml) | 18.4 ± 2.0 | 40.9 ± 19.6 | 0.0006 |
| Superficial cells (%) | 4.0 ± 9.1 | 34.1 ± 19.1 | 0.0002 |
| Vaginal pH (units) | 5.7 ± 1.3 | 4.7 ± 0.6 | 0.0283 |

[a]Values are mean ± SD.

**TABLE 2. Vaginal cultures**

| Microorganism | Pre-ERT (No. [%]) | Post-ERT (No. [%]) | *P* value[a] |
|---|---|---|---|
| Lactobacilli | 7 [47] | 9 [60] | NS |
| Any aerobe | 14 [93] | 13 [87] | NS |
| Any anaerobe | 7 [47] | 2 [13] | 0.05 |
| Anaerobic gram-negative rods | 6 [40] | 1 [7] | 0.035 |

[a]NS, not significant.

### RESULTS

The mean age of the subjects was 53.9 ± 8.6 years. Nine (60%) were sexually active, and 5 (33%) gave a history of douching. Throughout the course of the study, the subjects maintained a relatively constant level of sexual activity. No subject douched or had sexual intercourse within 3 days of having the vaginal cultures taken. No vaginal medications were used during the study period. None of the subjects were exposed to any antimicrobial agents. Seven of the 15 subjects had undergone hysterectomy.

Prior to ERT, all of the subjects exhibited serum FSH and estradiol levels consistent with ovarian failure. After 8 weeks of estrogen replacement, the sample mean serum estradiol levels were significantly increased, while serum FSH levels were suppressed. After the subjects received ERT, an increased superficial cell count was noted, reflecting the enhanced epithelialization of the vagina under the influence of estrogen. Finally, the mean vaginal pH was noted to be decreased significantly following ERT. These results are summarized in Table 1.

Detailed in Table 2 are the results of the vaginal cultures obtained before and after ERT. Although lactobacilli were among the most frequently isolated species both before and after estrogen replacement, the proportion of isolates did not significantly change following treatment. The nonenterococcal group D streptococci (5), *Staphylococcus epidermidis* (4), and gram-negative enterics (4) were also commonly isolated. No change was noted in the frequency with which the subjects tested positive for any aerobe. However, the prevalence of anaerobic isolates decreased considerably after ERT. The difference was most pronounced for the anaerobic gram-negative rods, which were isolated from 43% of the participants prior to treatment compared with only 7% after estrogen replacement. The majority [5/6 (83%)] of the anaerobic gram-negative rods disappearing on ERT were bile-resistant *Bacteroides* spp. [*B. capillosus, B. fragilis* (two), *B. ovatus* (two)].

Finally, the mean vaginal pH after ERT in lactobacilli-positive subjects was 4.4 ± 0.4 compared with 5.2 ± 0.3 in lactobacilli-negative subjects ($P = 0.02$). There was no difference between vaginal pH before ERT in lactobacilli-positive subjects (5.5 ± 1.3) compared with lactobacilli-negative subjects (5.9 ± 1.4) ($P = 0.65$). Although the subjects colonized with lactobacilli exhibited a consistently lower mean vaginal pH, this difference was only significant following estrogen replacement.

### DISCUSSION

Unlike previous investigations detailed in the literature, the present study utilized a single postmenopausal population, obtaining vaginal bacterial isolates before and after ERT and documenting the hormonal status at each step by serum FSH and estradiol levels and by serial examination of vaginal cytology. Consequently, each patient served as her own control, allowing a more direct appraisal of the effects of estrogen on the vaginal pH and microbiology.

These data confirm previous reports that a change in hormonal status is associated with changes in vaginal bacterial flora. In accord with the findings of Larsen et al., the serial cultures employed in this study revealed a decreased prevalence of anaerobic species, especially anaerobic gram-negative rods, following the administration of exogenous estrogen.[3] The number of aerobic isolates remained relatively constant.

The incidence of lactobacilli did not significantly change following estrogen replacement despite a documented increase in serum estrogen to levels sufficient to suppress FSH, supporting the theory that hormonal status alone plays an important independent role in the maintenance of a low vaginal pH.[5] These findings are consistent with an estrogen-induced increase in epithelial cell metabolism of glycogen, resulting in a lower vaginal pH. However, following ERT, the mean vaginal pH was significantly lower in lactobacilli-positive subjects. This finding suggests that lactobacilli contribute to a decrease in vaginal pH and raises the possibility that estrogen may in some way potentiate the effect of lactobacilli on vaginal pH. By increasing the carbohydrates available for metabolism to lactic acid by lactobacilli, ERT may help decrease vaginal pH without producing a commensurate change in the incidence of lactobacilli. The lower pH associated with ERT yields an environment less suitable to anaerobes.[6,7] In addition, estrogen may enhance the ability of lactobacilli to produce hydrogen peroxide, which may control the quantity of catalase-negative anaerobic microorganisms. Further investigation is needed in these areas.

This investigation is limited by the small number of subjects studied and by the brief time over which the subjects were followed. A decrease in vaginal pH should select for acidiphilic species; however, any increase in the isolation of acidophilic species first requires new colonization, a process that may take more time than the 8 weeks allowed in this study. In the small population utilized here, only three subjects had negative cultures for lactobacilli both before and after ERT. Given a larger number of such subjects, we might have been able to test for a significant change in mean vaginal pH in this subpopulation, thereby better demonstrating the effect of estrogen on vaginal pH independent of its effect on lactobacilli.

In summary, vaginal cultures obtained from postmenopausal women prior to the initiation of ERT were more likely to grow anaerobic bacteria when compared with vaginal cultures obtained from these same women after the initiation of ERT. ERT may potentiate the effect of lactobacilli on vaginal pH.

### REFERENCES

1. Tashjian JH, Coulan CB, Washington JA: Vaginal flora in asymptomatic women. Mayo Clin Proc 51:557–561, 1976.
2. Osborne NG, Wright RC, Grulin L: Genital bacteriology: A comparative study of premenopausal women with postmenopausal women. Am J Obstet Gynecol 135:195–198, 1979.

3. Larsen B, Goplerud CP, Petzold CR, Ohm-Smith MJ, Galask RP: Effect of estrogen treatment on the genital tract flora of postmenopausal women. Obstet Gynecol 60:20–24, 1982.
4. Lennette EH, Balows A, Hausler WJ, Shadomy HJ: Manual of Clinical Microbiology. 4th ed. Washington, DC: American Society for Microbiology, 1985.
5. Kienlin H: Die reaktion des vaginakelkrebs neugeborener. Zentralbl Gynak 50:644, 1926.
6. Brown WJ: Microbial ecology of the normal vagina. In Haafex ESE, Evans N (eds): The Human Vagina. New York, Elsevier/North Holland Biomedical Press, pp 407–422, 1978.
7. Paavonen J: Physiology and ecology of the vagina. Scand J Infect Dis Suppl 40:31–35, 1983.
